# Mesopic and Low-Contrast Visual Acuity Deficits in Retinitis Pigmentosa: Clinical Markers for Early Functional Impairment

**DOI:** 10.3390/jcm14165659

**Published:** 2025-08-10

**Authors:** Juan E. Cedrún-Sánchez, Francisco Javier Povedano-Montero, Eva Chamorro, Celia Sánchez-Ramos, María C. Puell

**Affiliations:** 1 Department of Optics and Optometry, Faculty of Optics and Optometry, Universidad Complutense de Madrid, 28037 Madrid, Spain; jcedrun@ucm.es (J.E.C.-S.); celiasr@opt.ucm.es (C.S.-R.); puellma@ucm.es (M.C.P.); 2 Applied Vision Research Group, Faculty of Optics and Optometry, Universidad Complutense de Madrid, 28037 Madrid, Spain; 3 Hospital Doce de Octubre Research Institute (i + 12), 28041 Madrid, Spain; 4 Clinical Research Department, Indizen Optical Technologies, 28002 Madrid, Spain; evachamorro@iot.es

**Keywords:** retinitis pigmentosa, low-contrast visual acuity, low-luminance visual acuity, visual acuity outcome measures, mesopic visual acuity

## Abstract

**Background:** Standard visual acuity (VA) is often preserved in early retinitis pigmentosa (RP), limiting its value as a marker of functional impairment. Alternative measures such as low-luminance deficit (LLD) and low-contrast deficit (LCD) may detect earlier changes in cone function. This study aimed to evaluate the diagnostic utility of these measures in RP patients under photopic and mesopic conditions. **Methods:** A prospective observational study was conducted on 57 RP patients and 54 age-matched controls. Binocular VA was assessed using ETDRS charts at 100% and 10% contrast under photopic (100 cd/m^2^) and mesopic (1 cd/m^2^) conditions. LLD and LCD scores were computed from VA differences across conditions. ROC curve analysis was used to determine diagnostic accuracy. **Results:** RP patients showed significant VA loss under reduced luminance and contrast (*p* < 0.001), independent of age. LLD under high contrast was reduced, while LLD under low contrast and LCD (both photopic and mesopic) were significantly higher than in controls. The mesopic LCD demonstrated the highest diagnostic capacity (AUC = 0.87), with a threshold of >13 ETDRS letters yielding optimal sensitivity and specificity. Unlike standard VA, mesopic LCD correlated with functional symptoms and was unaffected by age. **Conclusions:** Low-contrast VA under mesopic conditions is a simple, reproducible, and sensitive marker for early visual dysfunction in RP. A difference > 13 ETDRS letters may serve as a clinically relevant threshold for disease monitoring and early detection in retinal dystrophies.

## 1. Introduction

Retinal degenerations are a major cause of visual impairment and reduced quality of life. In acquired conditions such as age-related macular degeneration (AMD), which typically affects individuals over the age of 60, tasks such as reading, driving, and facial recognition become increasingly difficult [1]. In contrast, hereditary retinal diseases like retinitis pigmentosa (RP) often present earlier in life, impacting patients during their most productive years and significantly affecting occupational performance [2].

RP is a progressive, inherited retinal disorder that affects approximately 1 in 4000 individuals worldwide [3]. While most cases are non-syndromic, 20–30% of patients may exhibit associated systemic conditions. The disease is characterized by the gradual degeneration of photoreceptors, leading to progressive vision loss. Although there is currently no definitive cure, the field is rapidly evolving, with emerging therapeutic strategies such as gene therapy, stem cell transplantation, and optogenetics offering promising avenues for treatment [4].

The clinical course of RP is highly variable. Although onset commonly occurs in the second or third decade of life, both earlier and later presentations are observed due to extensive genetic heterogeneity—over 80 genes have been implicated in non-syndromic RP [5]. This variability complicates the definition of typical clinical features and progression patterns.

Night blindness often appears in adolescence as the earliest symptom, followed by concentric visual field constriction, reflecting primary rod dysfunction. Central vision tends to remain preserved until the later stages, when cone involvement becomes evident. As a result, standard visual acuity (VA) testing alone may not adequately reflect functional impairment, highlighting the need for alternative, sensitive visual function markers [6].

Several studies have explored VA performance under different contrast and luminance conditions. Lin et al. demonstrated that using a 2.0 log unit neutral density filter produces a significant and reproducible reduction in VA under mesopic lighting, similar to low-contrast testing [7]. These findings support the use of mesopic luminance levels between 0.75 and 1 cd/m^2^ for functional assessment.

ETDRS charts are available in multiple contrast levels (100%, 50%, 25%, 10%, 2.5%, and 1.25%), although testing protocols vary widely. It is well established that as contrast decreases, visual performance declines, especially in the presence of retinal disease. This enables early detection of visual dysfunction, albeit with increased test failure rates at very low contrast. For example, Wood et al. reported that 38% of patients with choroideremia could not complete testing at 1.25% contrast, and 21% failed at 2.5% [8].

To balance clinical sensitivity with feasibility, the 10% contrast chart is widely used. It better reflects real-world conditions and maintains adequate test reliability. Regan et al. found that charts with 10% and 25% contrast provided the best sensitivity to functional changes, although the 25% chart elicited minimal VA differences [9,10]. Hence, the 10% chart is considered more appropriate for detecting early visual decline in retinal disease.

Although comprehensive structural and functional assessments such as OCT, microperimetry, and electrophysiology offer detailed insights, their implementation is often constrained by cost, technical complexity, and limited accessibility—particularly in early screening or routine follow-up. While these methods are not directly comparable to visual acuity tests in terms of diagnostic objectives, they are commonly reserved for confirmatory diagnosis or advanced disease staging.

In contrast, low-luminance and low-contrast VA testing are simple, inexpensive, and widely applicable tools. They offer advantages in terms of accessibility and ease of use for preliminary detection of functional changes. In the context of the Spanish healthcare system, access to advanced diagnostic tests may be limited by long waiting times in public services or involve significant out-of-pocket expenses in private practice, which reinforces the relevance of accessible, non-invasive functional assessments.

The present study aimed to evaluate the utility of these measures in RP patients, assess the influence of age and disease stage, and compare findings with previously reported results in other retinal pathologies under similar testing conditions.

## 2. Materials and Methods

This was a prospective, cross-sectional, analytical, observational study conducted in individuals diagnosed with retinitis pigmentosa (RP) and a group of healthy controls matched for age. RP patients were recruited from the Asociación Retina Madrid (Madrid, Spain), and controls were selected from the general population with no history of ocular disease.

Inclusion criteria for the RP group included a confirmed diagnosis of RP and best-corrected binocular visual acuity better than 1.25 logMAR. Participants were excluded if they had any additional ocular pathology (e.g., glaucoma, significant cataracts, diabetic retinopathy), or systemic diseases with known ocular involvement (e.g., uncontrolled diabetes, neurological disorders). The same exclusion criteria were applied to the control group to ensure comparability.

The study adhered to the principles of the Declaration of Helsinki and was approved by the Ethics Review Board of Hospital Clínico San Carlos (Madrid, Spain; CEIC). Written informed consent was obtained from all participants after providing detailed information about the study protocol.

### 2.1. Procedures

All participants underwent a comprehensive optometric examination to determine their best optical correction and to confirm eligibility criteria. Early Treatment Diabetic Retinopathy Study (ETDRS) charts and results were recorded in logMAR units. Anterior segment structures were assessed using a slit-lamp biomicroscope (Topcon SL-D2, Tokyo, Japan), and any anomalies potentially affecting visual function were noted.

RP patients were also asked to report disease-related visual symptoms. A structured questionnaire was used to assess the presence and age of onset of eight common symptoms: night blindness, visual field constriction, reduced visual acuity, diminished contrast sensitivity, color vision alterations, dark adaptation problems, photophobia, and glare sensitivity.

VA was measured using both high-contrast (100%) and low-contrast (10%) ETDRS charts. The order of contrast conditions and lighting levels was randomized. Testing was performed under the following conditions:

- Photopic conditions 100 cd/m^2^.
- Mesopic conditions 1 cd/m^2^, obtained by placing a 2.0 log unit neutral density filter (Rosco e-colour 211; Rosco-Ibérica S.A., Madrid, Spain) over the backlit ETDRS cabinet panel.

All measurements were taken binocularly. Control participants underwent 10 min of dark adaptation prior to mesopic testing, while RP patients required 20 min due to their increased sensitivity to glare and slower dark adaptation.

Luminance levels were verified using the Mavolux 5032B USB photometer (Gossen, Nürnberg, Germany).

VA scores in logMAR units were converted to ETDRS letter scores (also known as visual acuity rating, VAR) using the following equation:

VAR = 100 − (50 × logMAR)(1)

All participants completed a standardized visual acuity assessment protocol, which included high-contrast visual acuity under photopic conditions (HCVAP, also referred to as standard VA), 10% low-contrast visual acuity under photopic conditions (10% LCVAP), high-contrast visual acuity under mesopic conditions (HCVAM), and 10% low-contrast visual acuity under mesopic conditions (10% LCVAM).

To quantify visual impairment under low-luminance and -contrast conditions, the following indices were calculated:

Low-luminance deficit with high contrast (LLD_HC_): calculated as the difference between high-contrast visual acuity under photopic and mesopic conditions:

(2) $${LLD}_{HC}={HCVA}_{P}-{HCVA}_{M}$$

Low-luminance deficit with low contrast (LLD_LC_): defined as the difference between 10% low-contrast visual acuity under photopic and mesopic conditions:

(3) $${LLD}_{LC}={10\%\;LCVA}_{P}-{10\%\;LCVA}_{M}$$

Low-contrast deficit (LCD): determined by subtracting 10% low-contrast visual acuity from high-contrast visual acuity under both lighting conditions as follows:

Under photopic conditions:

(4) $${LCD}_{P}={HCVA}_{P}-{10\%\;LCVA}_{P}$$

Under mesopic conditions:

(5) $${LCD}_{M}={HCVA}_{M}-{10\%\;LCVA}_{M}$$

These indices were used to characterize the impact of luminance and contrast reduction on visual performance.

### 2.2. Statistical Analysis

Non-parametric comparative statistical analyses were applied due to the non-normal distribution of the data. All statistical procedures were conducted using Statgraphics 19-X64 (Statpoint Technologies, Inc., The Plains, VA, USA).

The ability of individual variables and the composite discriminant function to differentiate between eyes with retinitis pigmentosa (RP) and healthy control eyes was assessed by comparing the areas under the receiver operating characteristic (ROC) curves (AUCs). A test was considered valid when the AUC exceeded 0.70. For the ROC curve analysis, Sigmaplot 11 software (Systat Software, Inc., San Jose, CA, USA) was used. Statistical significance was set at *p* < 0.05.

## 3. Results

A total of 57 individuals diagnosed with retinitis pigmentosa (RP) were enrolled in the study (median age: 48 years; range: 23–71 years), of whom 31 were female and 26 males. Additionally, an age-matched control group comprising 54 healthy individuals was recruited (median age: 30 years; range: 20–87 years), including 34 females and 20 males. No significant age difference was found between the groups (Mann–Whitney U test, *p* = 0.1701). Demographic characteristics of all participants are presented in Table 1.

Control participants were recruited from students, their family members, and acquaintances attending the University Clinic of Optometry at the Complutense University of Madrid. Individuals with RP were recruited through the Asociación Retina Madrid (Madrid, Spain). All participants, regardless of group assignment, were able to read letters on each of the four visual acuity (VA) tests.

**Figure 1:**
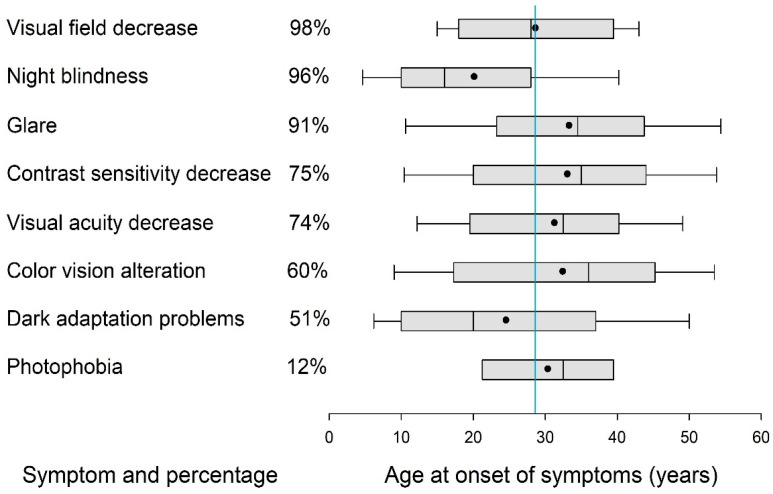
Most frequent visual symptoms in RP patients showing percentage reporting each symptom and age of onset; dots represent means, and the blue line indicates the average symptom onset.

Figure 1 shows the self-reported vision-related symptoms of the patients with RP. The most frequently reported symptoms were visual field constriction (98%), night blindness (96%), and glare sensitivity (91%). The average age of onset for vision-related symptoms was 29 years. Night blindness was typically the first symptom to appear, with a mean onset age of 20 ± 13 years.

In patients with retinitis pigmentosa (RP), the mean visual field extent was 11.49 ± 5.21 degrees (minimum = 4°, maximum = 25°), indicating a marked reduction consistent with peripheral field constriction. Regarding color vision, as assessed with the Farnsworth D-15 test, a tritan defect was observed in 43% of RP patients, protan defects in 2%, complete color blindness in 4%, and nonspecific dyschromatopsia in 9%.

In contrast, of the healthy controls, 4% showed protan defects and 23% showed mild deutan anomalies. In the RP group, color vision impairment was present across all age ranges. However, in the control group, color vision alterations were found only in individuals over 65 years of age, and were generally mild, except in three participants older than 69 years who exhibited a pronounced deutan defect (representing 6% of the control sample).

These findings suggest that color vision abnormalities in RP are primarily disease-related and not age-dependent, whereas in healthy individuals, age-related dyschromatopsia—particularly of the deutan type—may begin to emerge after the age of 65.

Visual acuity scores under varying lighting and contrast conditions are detailed in Table 2. As expected, VA declined with reduced contrast and, more markedly, under mesopic conditions. These declines were significantly greater in RP patients than in controls.

**Figure 2:**
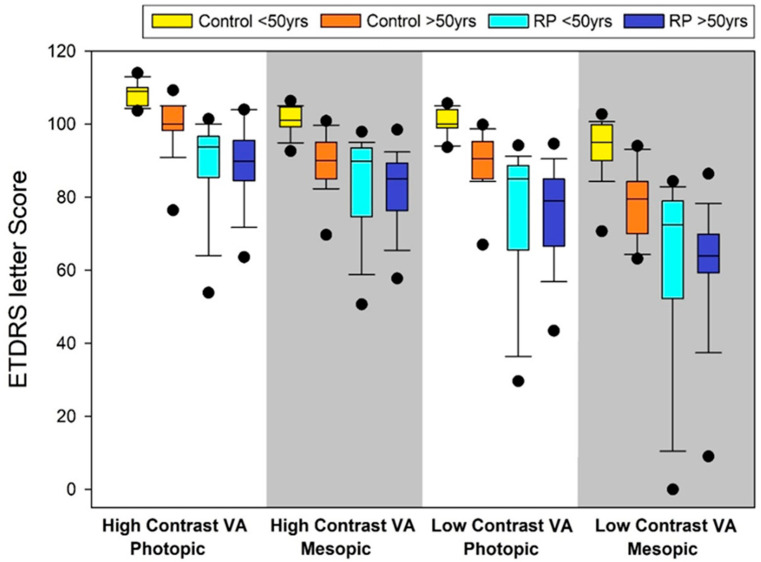
Box plots of the four visual acuity tests comparing RP patients and controls. ETDRS: Early Treatment Diabetic Retinopathy Study scores; VA: visual acuity.

To assess the influence of age, participants were stratified into two subgroups: under 50 years and 50 years or older. In the healthy controls, older participants had consistently lower VA across all conditions, suggesting age-related decline. In contrast, RP patients exhibited similarly reduced VA across age groups, indicating that disease severity had a greater impact than age alone. Figure 2 shows the distribution of ETDRS letter scores for the four VA tests in RP and control subjects.

Deficit scores derived from the visual acuity tests revealed further between-group differences (Table 3). RP patients showed significantly lower scores for the low-luminance deficit under high contrast (*p* < 0.001), and higher scores for the low-luminance deficit under low contrast, as well as for contrast deficits under both photopic and mesopic conditions (*p* < 0.001 for all). Of all the test conditions, the 10% low-contrast mesopic test yielded the largest performance gap between groups.

The magnitude of the between-group differences was as follows: three letters for the high-contrast low-luminance deficit, six letters for the low-contrast low-luminance deficit, and three letters for the photopic-contrast deficit (all *p* < 0.001). The greatest difference was found in the mesopic-contrast deficit, with RP patients scoring on average 10 letters fewer than controls (*p* < 0.001), equivalent to two lines on the ETDRS chart.

**Figure 3:**
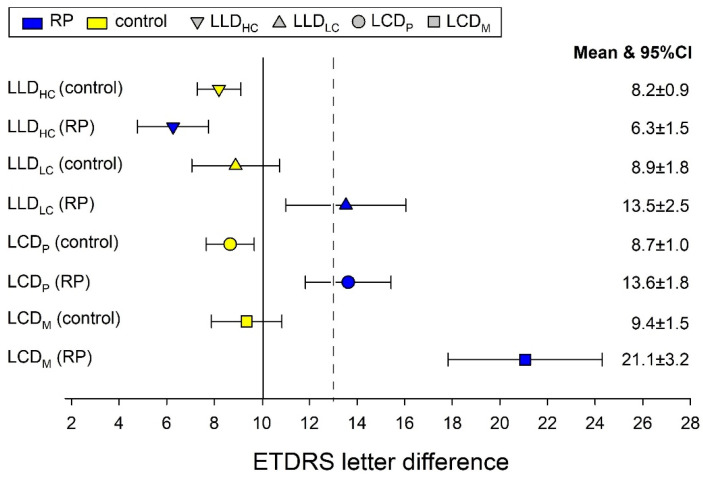
Forest plot of luminance and contrast deficits in controls and RP patients. The dashed line marks the upper 95% confidence limit of 13 ETDRS letters.

To illustrate the differences in functional visual performance between patients with retinitis pigmentosa (RP) and healthy controls, a summary forest plot was generated incorporating all calculated indices (Figure 3). This plot displays mean values and 95% confidence intervals for high-contrast and low-contrast low-luminance deficits (LLD-HC, LLD-LC) and contrast deficits under both photopic and mesopic conditions (LCD-P, LCD-M). In the control group, all indices remained below the clinical threshold of 13 ETDRS letters, whereas in the RP group, the LCD-M reached an average of 21.1 letters—emerging as the most prominent and sensitive parameter. The vertical dashed line marks the 13-letter upper 95% confidence limit derived from normative data, visually emphasizing the separation between physiological and pathological function.

**Figure 4:**
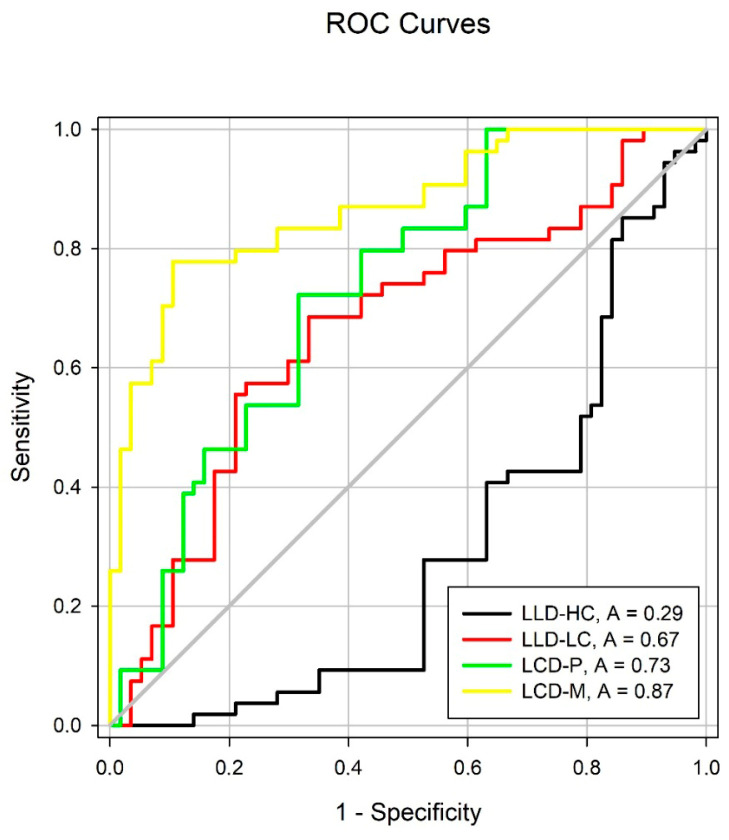
Receiver operating characteristic (ROC) curves for LLDHC, LLDLC, LCDP, and LCDM. LCDM (yellow) showed the highest diagnostic accuracy (AUC = 0.87), followed by LCDP (green). LLDHC (black) demonstrated poor diagnostic capacity. AUC: area under the curve.

The diagnostic performance of the low-luminance and contrast-derived indices—LLDHC, LLDLC, LCDP, and LCDM—was evaluated using receiver operating characteristic (ROC) curves, as shown in Table 4 and Figure 4. Of the four indices, only LCDP and LCDM showed area under the curve (AUC) values above the threshold of 0.70, indicating acceptable discriminative ability. The highest diagnostic accuracy was observed for LCDM, with an AUC of 0.87 ± 0.03 (*p* < 0.0001), suggesting strong capability to distinguish RP eyes from healthy controls and a low false positive rate.

In contrast, LLDHC and LLDLC did not reach the 0.70 threshold (AUC = 0.29 and 0.67, respectively), indicating limited or no diagnostic value in this sample. These findings confirm that deficit under mesopic conditions (LCDM) is the most robust functional parameter for early detection of RP.

## 4. Discussion

This study demonstrates that standard high-contrast visual acuity testing is insufficient for detecting early functional loss in patients with retinitis pigmentosa (RP), whereas assessments under low-luminance and low-contrast conditions reveal substantial deficits. Among the parameters tested, mesopic deficit (LCDM) showed the highest sensitivity to early retinal dysfunction, highlighting its potential as a clinical biomarker. These results support the incorporation of contrast- and luminance-based VA testing into routine RP assessments, particularly in early or ambiguous stages.

In RP patients, high-contrast visual acuity under mesopic conditions (HCVAM) showed minimal reduction compared to standard VA, with a mean difference of only two letters. This may explain why individuals with RP, despite experiencing pronounced glare, often report greater comfort in low-light environments. Visual discrimination is relatively preserved when contrast is high, even under mesopic conditions, but deteriorates markedly when contrast is reduced.

Under photopic conditions, low-contrast VA declined more in the RP group than in controls, likely due to increased intraocular light scatter. This is mainly attributable to the cornea and crystalline lens. Previous studies have suggested that the posterior capsule in RP patients contributes significantly to scatter, possibly due to accumulation of inflammatory by-products [11,12,13,14].

Additional optical degradation in RP arises from depigmentation of the retinal pigment epithelium (RPE), especially in the mid-peripheral retina, due to melanin loss [15]. Melanin plays an important optical role by reducing intraocular light scatter and enhancing contrast sensitivity. Its concentration is highest in the peripheral RPE, and it declines with age—an effect exacerbated in RP [16,17,18,19].

All tests in our study were performed using standard ophthalmic equipment and protocols that are clinically accessible and time-efficient. The repeatability of low-contrast and low-luminance VA is comparable to that of standard VA. Barrio et al. suggested a threshold of ≥five letters as clinically significant when using 100% and 10% ETDRS charts under mesopic conditions [20]. Wood et al. reported repeatability coefficients of six letters in healthy controls and seven letters in patients with RP or choroideremia [21].

Low-luminance and low-contrast VA reflect central cone function in RP, as peripheral rods are affected earlier. A measurable reduction in VA under diminished contrast or luminance may therefore indicate early central cone involvement [22,23]. These changes can be quantified through luminance deficit (LLD) and contrast deficit (LCD), which compare standard VA with VA under challenging conditions [8,24].

Wood et al. reported that the average LLD in healthy subjects is 10 ETDRS letters, with a 95% confidence upper limit of 13 letters using a 2.0 log unit neutral density filter [25]. A score above 13 letters may thus indicate early retinal dysfunction and be useful for clinical monitoring.

In our RP cohort, LLD values remained below 13 letters, consistent with Wood et al.’s findings in RP and choroideremia [21]. Similar results have been found in other diseases such as PXE and macular telangiectasia type 2 [26,27].

In AMD, studies show that LLD values typically remain below 13 letters in early stages and exceed this threshold in intermediate AMD [24,28,29,30,31,32], supporting the diagnostic value of this cutoff.

To contextualize our findings, we reviewed LLD and LCD values from studies using comparable protocols in other retinal diseases. Supplementary Figures S1 and S2 summarize these data, reinforcing the clinical utility of the 13-letter threshold, which is exceeded in diseases such as choroideremia and PXE. In our cohort, mesopic LCD was well above this threshold, supporting its relevance as a functional marker.

In the healthy controls, previous studies using 100% and 10% contrast ETDRS charts reported LCD values of 10 letters, with a maximum of 13 letters [33,34]. Puell et al. found LCD below 13 letters in early AMD [34], while Tomita et al. reported an average of 24 letters in advanced AMD [6]. Higher LCDs have also been observed in diabetic retinopathy (DR) and diabetic macular edema (DME) [33].

In our RP patients, LCD exceeded 13 letters in photopic conditions and reached 21 under mesopic lighting. Compared to controls, this represented differences of 5 and 12 letters, respectively.

Age-related changes such as reduced cone density, inner retinal thinning, and lens opacities exacerbate declines in low-contrast and low-luminance VA compared to standard VA [35,36,37,38,39].

Although no formal anatomical or functional classification of RP stage was applied, all patients included in this study had preserved central vision (better than 1.25 logMAR) and were able to complete all visual tests under both lighting and contrast conditions. This implicitly limited the cohort to cases with mild or moderate central involvement. As our functional outcomes are based on binocular central visual acuity, it is unlikely that patients with very advanced disease stages were represented. Although the extent of visual field loss was recorded globally in degrees, no formal correlation analysis was conducted between field parameters and VA outcomes due to the absence of standardized perimetric data. Future studies incorporating structured staging systems (e.g., OCT-based or visual field protocols) may help refine the clinical interpretation and applicability of these measures across the full spectrum of RP severity.

When stratifying by age (<50 vs. ≥50 years), we observed that healthy controls experienced more pronounced age-related losses under challenging conditions. In contrast, RP patients showed minimal age-related variation: LLD and LCD scores remained largely stable across age groups. For instance, LCDM differed by only one letter between age groups in both controls and RP.

These findings suggest that age has limited impact on LLD and LCD in RP, unlike other conditions where aging is a major factor. Further research is needed to confirm these observations, particularly in systemic diseases like diabetes where age plays a more prominent role.

Another limitation of the present study is the absence of structural correlation using optical coherence tomography (OCT). Although our primary aim was to evaluate the diagnostic utility of mesopic and low-contrast visual acuity under routine clinical conditions, combining these functional measures with morphological parameters—such as ellipsoid zone integrity or central retinal thickness—would have enhanced the validation of our approach. Future studies incorporating multimodal imaging may help establish more robust structure–function relationships in RP and better define the clinical significance of early visual deficits.

## 5. Conclusions

In patients with retinitis pigmentosa (RP), age does not significantly affect standard visual acuity or low-luminance and low-contrast visual performance.

A threshold of more than 13 ETDRS letters in contrast deficit (LCD) and luminance deficit (LLD) can serve as a useful functional marker for disease monitoring in ocular pathologies. However, these indices should be interpreted alongside absolute VA values under reduced conditions, as dissociation may occur between LLD/LCD and standard VA, particularly in diseases with localized central involvement. This discrepancy reflects the fact that standard VA primarily assesses foveal function, whereas LLD and LCD capture broader macular activity.

In our RP cohort, LLD values did not significantly differ from controls, while LCD values were notably increased, particularly under mesopic lighting. These findings support the use of mesopic LCD as a more sensitive marker of functional impairment in RP.

This study has several limitations that should be acknowledged. First, its cross-sectional design precludes any conclusions about causality or longitudinal progression. Second, while the clinical sample was well characterized, the sample size may limit generalizability to other genetic subtypes of RP or more advanced stages. In addition, comparisons with other retinal diseases were based on literature data rather than direct cohorts, introducing potential methodological differences. Finally, no structural imaging (e.g., OCT or autofluorescence) was performed to correlate functional outcomes with anatomical changes.

Further research is warranted to assess the generalizability of these findings in other retinal dystrophies and to explore their prognostic value in broader clinical settings.

## Figures and Tables

**Table 1:** Subject’s demographics. Median and interquartile range (IQR).

	Control	RP	Mann–Whitney U Test
No. of subjects (n)	54	57	
Age groups (<50 years, >50 years)	(32, 22)	(32, 25)	0.2327
Age, median (range)	30.0 (20, 87)	48.0 (23, 71)	0.1701
Female, n (%)	34 (63%)	31 (54.4%)	0.9682
Spherical Equivalent RE (D)	0.00 (−2.25, 0.00)	−0.38 (−1.13, 0.25)	0.3135
Spherical Equivalent LE (D)	−0.38 (−2.88, 0.00)	−0.13 (−0.63, 0.75)	0.0073
Stereopsis (arcseconds)	60 (60, 120)	240 (120, 1000)	<0.0001

**Table 2:** Subjects’ demographics, v acuity scores under different conditions.

	Control (n = 54)	RP (n = 57)	Mann–Whitney U Test
**ETDRS letter score**			
**High-contrast VA** **Photopic (HCVA_P_)**	105 (103, 109)	89 (85, 96)	<0.001
**High-contrast** **VA Mesopic (HCVA_M_)**	99 (90, 102)	87 (75, 91)	<0.001
**10% Low-contrast** **VA photopic (10%** **LCVA_P_)**	99 (93, 102)	80 (67, 87)	<0.001
**10% Low-contrast** **VA mesopic (10%** **LCVA_M_)**	90 (80, 96)	67 (59, 77)	<0.001

**Table 3:** Visual acuity and the calculated low-luminance and low-contrast difference results. Median and interquartile range (IQR).

	Control (*n* = 54)	RP (*n* = 57)	Mann–Whitney U Test
**ETDRS letter difference score**			
**Low-luminance deficit with** **high contrast (LLD_HC_)**	8.0 (5.0, 10.0)	4.9 (4.0, 7.4)	<0.001
**Low-luminance deficit with** **low contrast (LLD_LC_)**	6.0 (4.0, 12.0)	11.8 (7.6, 16.2)	<0.001
**Low-contrast deficit in** **photopic (LCD_P_)**	9.0 (5.0, 11.0)	12.0 (9.7, 16.8)	<0.001
**Low-contrast deficit in** **mesopic (LCD_M_)**	8.0 (5.5, 12.0)	18.0 (15.0, 24.0)	<0.001

**Table 4:** Diagnostic performance of visual function indices for detecting retinitis pigmentosa. Data are expressed as an area under the ROC curve (AUC ± standard error), with corresponding 95% confidence intervals and *p* values.

Variable	Control vs. RP AUC
Low-luminance deficit with high contrast (LLD_HC_)	
Mean ± standard error	0.29 ± 0.05
95% confidence interval	0.20, 0.39
*p* value	>0.05
Low-luminance deficit with low contrast (LLD_LC_)	
Mean ± standard error	0.67 ± 0.05
95% confidence interval	0.56, 0.77
*p* value	0.0025
Low-contrast deficit in photopic (LCD_P_)	
Mean ± standard error	0.73 ± 0.05
95% confidence interval	0.63, 0.82
*p* value	<0.0001
Low-contrast deficit in mesopic (LCD_M_)	
Mean ± standard error	0.87 ± 0.03
95% confidence interval	0.80, 0.94
*p* value	<0.0001

AUC = area under the ROC curve. Data are expressed as areas under the receiver operating characteristic (ROC) curve.

## Data Availability

The data supporting the conclusions of this study are available upon request from the corresponding author. Due to ethical and privacy restrictions, the data are not publicly accessible.

## Supplementary Materials

The following supporting information can be downloaded at https://www.mdpi.com/article/10.3390/jcm14165659/s1. Figure S1: Forest plot of low-luminance deficit (LLD) across ten studies (640 subjects) using the ETDRS chart and a 2.0 log unit neutral density filter under mesopic conditions in different retinal pathologies. Figure S2: Forest plot of low-contrast deficit (LCD) across four studies (416 subjects) using the ETDRS chart with 100% and 10% contrast under photopic and mesopic conditions in various retinal diseases.

## Abbreviations

The following abbreviations are used in this manuscript:

VA	Visual acuity
RP	Retinitis pigmentosa
LLD	Low-luminance deficit
LCD	Low-contrast deficit
AMD	Age-related macular degeneration
HCVAP	High-contrast visual acuity under photopic conditions
HCVAM	High-contrast visual acuity under mesopic conditions
LCVAP	Low-contrast visual acuity under photopic conditions
LCVAM	Low-contrast visual acuity under mesopic conditions
LLDHC	Low-luminance deficit with high contrast
LLDLC	Low-luminance deficit with low contrast
HCVAP	High-contrast VA photopic
HCVAM	High-contrast VA mesopic
RPE	Retinal pigment epithelium
OCT	Optical coherence tomography
DR	Diabetic retinopathy
DME	Diabetic macular edema
CHM	Choroideremia
PXE	Pseudoxanthoma elasticum
MacTel	Macular telangiectasia
ETDRS	Early Treatment Diabetic Retinopathy Study
ROC	Receiver operating characteristic
AUC	Area under the curve
VAR	Visual acuity rating
ND	Neutral density
