# Supplementary material for: Mesopic and Low-Contrast Visual Acuity Deficits in Retinitis Pigmentosa: Clinical Markers for Early Functional Impairment
Source: J Clin Med. 2025 Aug 10;14(16):5659. doi: 10.3390/jcm14165659 (PMC12386862; doi:10.3390/jcm14165659)
Supplement: Supplementary file 1 [file jcm-14-05659-s001.zip › jcm-3796805-supplementary.pdf]

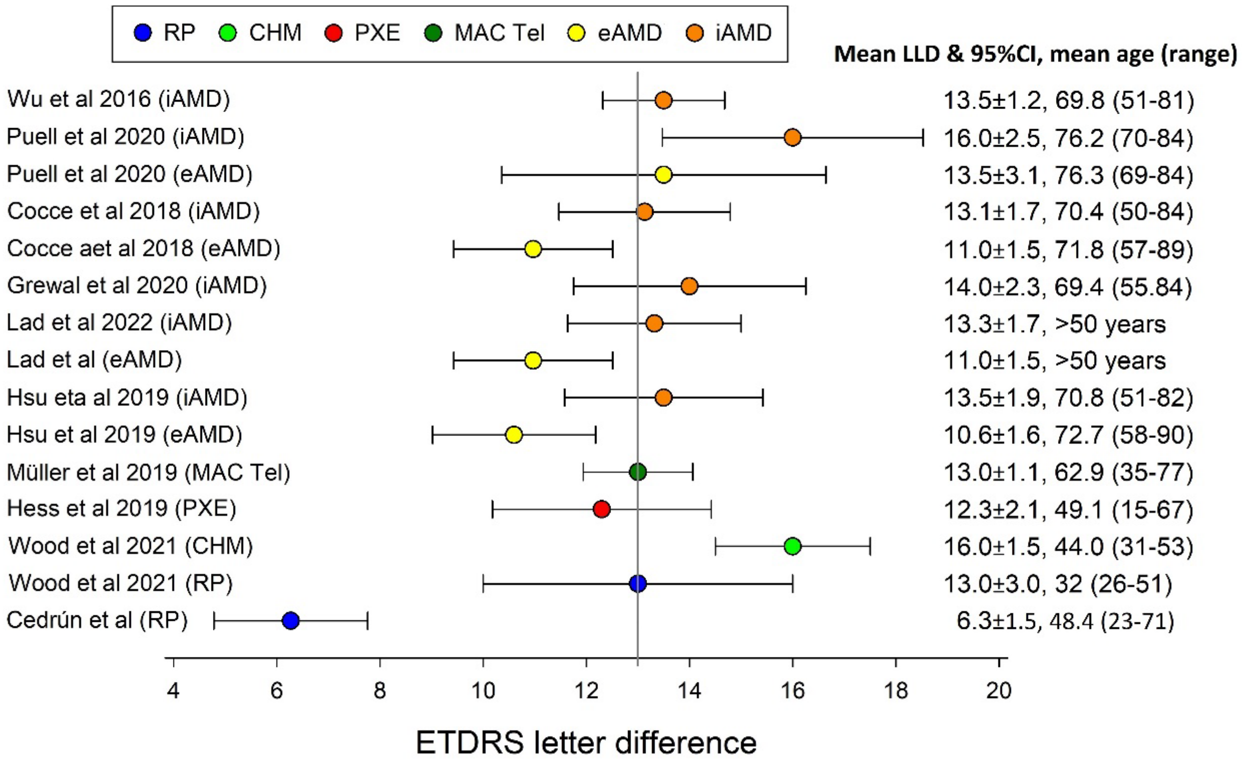

Figure S1. Forest plot for low luminance deficit (LLD) across ten studies (640 subjects) with different retinal pathologies using the ETDRS chart and a 2.0 log unit neutral density (ND) filter to measure visual acuity under light conditions mesopic. The solid gray line denotes the 95% confidence upper limit of 13 ETDRS letters found in studies with healthy subjects. Pathologies: retinitis pigmentosa (RP), choroideremia (CHM), pseudoxanthoma elasticum (PXE), macular telangiectasia type 2 (MAC Tel), early AMD (eAMD) and intermediate AMD (iAMD).

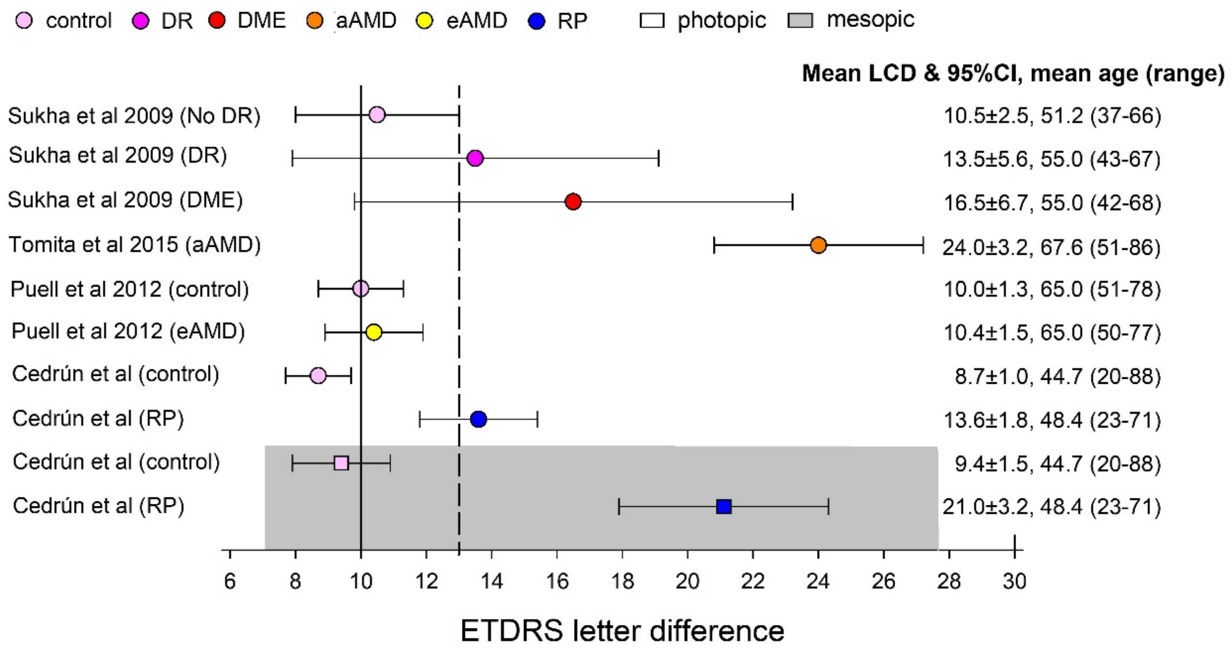

Figure S2. Forest plot for low contrast deficit (LCD) across four studies (416 subjects) with different retinal pathologies using the ETDRS chart with 100% and 10% contrast to measure visual acuity in photopic light conditions (light zone) and mesopic (grey zone). The solid line denotes the upper LCD normal limit and the dashed line indicates the 95% confidence upper limit of 13 ETDRS letters. Pathologies: diabetics without retinopathy (No DR), diabetic retinopathy (DR), diabetic macular edema (DME), early AMD (eAMD), advanced AMD (aAMD), retinitis pigmentosa (RP) and healthy control group.
